# Morphological determination of localization and function of Golgi proteins

**DOI:** 10.52601/bpr.2024.240008

**Published:** 2024-04-30

**Authors:** Yusheng Xing, Yannan Jian, Xiaodan Zhao, Yue Zhang, Zhenqian Zhang, Xing Zhang, Xiaoyan Zhang

**Affiliations:** 1 College of Biomedicine and Health, College of Life Science and Technology, Huazhong Agricultural University, Wuhan 430070, China

**Keywords:** Golgi apparatus, Golgi fragmentation, Immunofluorescence, Super-resolution microscopy, Electron microscopy, Lectin staining, Glycosylation

## Abstract

In animal cells, the Golgi apparatus serves as the central hub of the endomembrane secretory pathway. It is responsible for the processing, modification, and sorting of proteins and lipids. The unique stacking and ribbon-like architecture of the Golgi apparatus forms the foundation for its precise functionality. Under cellular stress or pathological conditions, the structure of the Golgi and its important glycosylation modification function may change. It is crucial to employ suitable methodologies to study the structure and function of the Golgi apparatus, particularly when assessing the involvement of a target protein in Golgi regulation. This article provides a comprehensive overview of the diverse microscopy techniques used to determine the specific location of the target protein within the Golgi apparatus. Additionally, it outlines methods for assessing changes in the Golgi structure and its glycosylation modification function following the knockout of the target gene.

## INTRODUCTION

The Golgi apparatus plays a vital role in the endomembrane transport system of eukaryotic cells. Its discovery dates back to 1898 when Camillo Golgi, an Italian physician and cytologist, observed an “internal reticular apparatus” in Purkinje cerebellar cells using the silver nitrate staining method (Mazzarello and Bentivoglio 1998). This structure was subsequently identified in diverse eukaryotic cell types and designated as the Golgi apparatus. Despite a history spanning over 125 years, the existence of the Golgi apparatus was a subject of intense debate among cell biologists for half of that time. It was not until the 1950s that the Golgi apparatus was confirmed as an independent membranous organelle with the widespread use of electron microscopy. Presently, it is known that the Golgi receives proteins and lipids from the endoplasmic reticulum (ER), orchestrating subsequent processing, packaging, and deliberate transport to diverse cellular destinations, including lysosomes, endosomes, the plasma membrane, or the extracellular space. Thus, the Golgi apparatus actively participates in frequent material exchange with other organelles within the endomembrane system, occupying a pivotal position within the cellular secretion pathway.

In contrast to the ER, which comprises of continuous network of interconnected tubules and sacs throughout the cytoplasm (Sun *et al.* 2024), the Golgi apparatus exhibits a unique structural composition. It consists of stacked, flattened membrane sacs termed cisternae (Zhang and Wang 2020). This arrangement imparts a distinct cis-to-trans polarity within the Golgi stack. As a result, the Golgi apparatus is divided into functionally specialized regions, specifically, the *cis-*Golgi network (CGN), *cis*-Golgi, *medial-*Golgi, *trans*-Golgi, and *trans-*Golgi network (TGN) (Short and Barr 2000). Each region plays distinct roles in the processing, modification, and sorting of cargo molecules passing through the Golgi.

The CGN is located closest to the ER and acts as the entry point for vesicles carrying newly synthesized proteins and lipids. In the ER, many proteins are modified by the addition of carbohydrate chains in a process called glycosylation (Zhang and Wang 2016). This modification is crucial for the structural integrity and functional diversity of the proteins. Upon arriving at the CGN and *cis-*Golgi, these sugar chains are first trimmed. Vesicles carrying modified proteins and lipids are subsequently transported to *medial-* and *trans-*Golgi cisternae, where additional glycosylation and other post-translational modifications take place. These include phosphorylation, sulfation, or proteolytic cleavage. The TGN acts as a sorting station; it packs proteins into transport vesicles and directs them to their final destinations, including other organelles such as lysosomes or endosomes, the plasma membrane, or the extracellular space (Guo *et al.* 2014). Thus, this organized arrangement allows for efficient compartmentalization and organization of various enzymes and molecules involved in protein processing, glycosylation, and vesicle trafficking.

Moreover, most mammalian cells typically possess multiple Golgi stacks, which can laterally connect to form a ribbon-like structure near the cell nucleus. Maintaining the structural integrity of the Golgi stacks and ribbon is crucial for its precise functioning. Several well-characterized proteins, such as Golgi matrix proteins (GRASPs and Golgins), play regulatory roles in the formation of the Golgi stacks and ribbon as well as vesicle binding on the cytoplasmic side of the Golgi membrane (Witkos and Lowe 2015; Zhang and Wang 2020).

The Golgi apparatus is a highly dynamic organelle capable of adapting its structure and functions in response to external stimuli. For example, during apoptosis, multiple Golgi structural proteins are cleaved by the caspase family enzymes, leading to Golgi fragmentation and cell apoptosis (Li *et al.* 2019). Under pathological conditions, such as tumors or neurodegenerative diseases, the Golgi apparatus undergoes fragmentation and protein glycosylation is significantly disrupted (Bajaj *et al.* 2022; Bui *et al.* 2021; Joshi *et al.* 2014). Therefore, studying these morphological changes, molecular mechanisms, and corresponding functional alterations in the Golgi apparatus can provide insights into disease development, diagnosis and treatment.

Researchers have employed a variety of techniques to understand the structure and function of the Golgi apparatus. These include cellular imaging for morphology visualization, biochemical approaches for proteins and lipids isolation and characterization, and genetic manipulation to determine the specific roles played by certain proteins in Golgi structure and functions. This article aims to summarize commonly used assays in Golgi morphology-related studies, offering primary guidance to researchers interested in studying the Golgi apparatus.

## THE MORPHOLOGY OF THE GOLGI VARIES AMONG DIFFERENT CELL LINES

Immunofluorescence microscopy is a commonly employed technique for visualizing the Golgi apparatus. By utilizing distinct Golgi-localized markers, such as GM130 (a *cis*-Golgi marker) and B4GALT1(a *trans*-Golgi marker) as detailed in Table 1, researchers were able to observe a condensed perinuclear region in HeLa cells and many other cell types.

**Table 1:** Commonly used Golgi markers (Bui *et al.* 2023)

Subcellular localization	Protein name	Source and Cat#
Cis-Golgi	GOLGA2/GM130	Proteintech, 11308-1-AP
		Proteintech, 66662-1-IG
	GOLGA3/Golgin-160	Proteintech, 21193-1-AP
	P115	Proteintech, 13509-1-AP
	GORASP1/GRASP65	
	GPP130	
	MGAT1	
	MAN1A1	
	Syntaxin 5	
Medial/Trans Golgi	GORASP2/GRASP55	Proteintech, 10598-1-AP
	MAN2A1	
	GOLGA5/Golgin-84	
	Golgin-45	
Trans Golgi	GCC1/GCC88	Proteintech, 16271-1-AP
	GOLGA1/Golgin-97	Proteintech, 12640-1-AP
	B4GALT1	Abclonal, A8546
	ST6GAL1	
	GCC185	
	GOLGA4/Golgin-245	
Trans Golgi network	TGN46	Bio-Rad, AHP500

**Figure 1:**
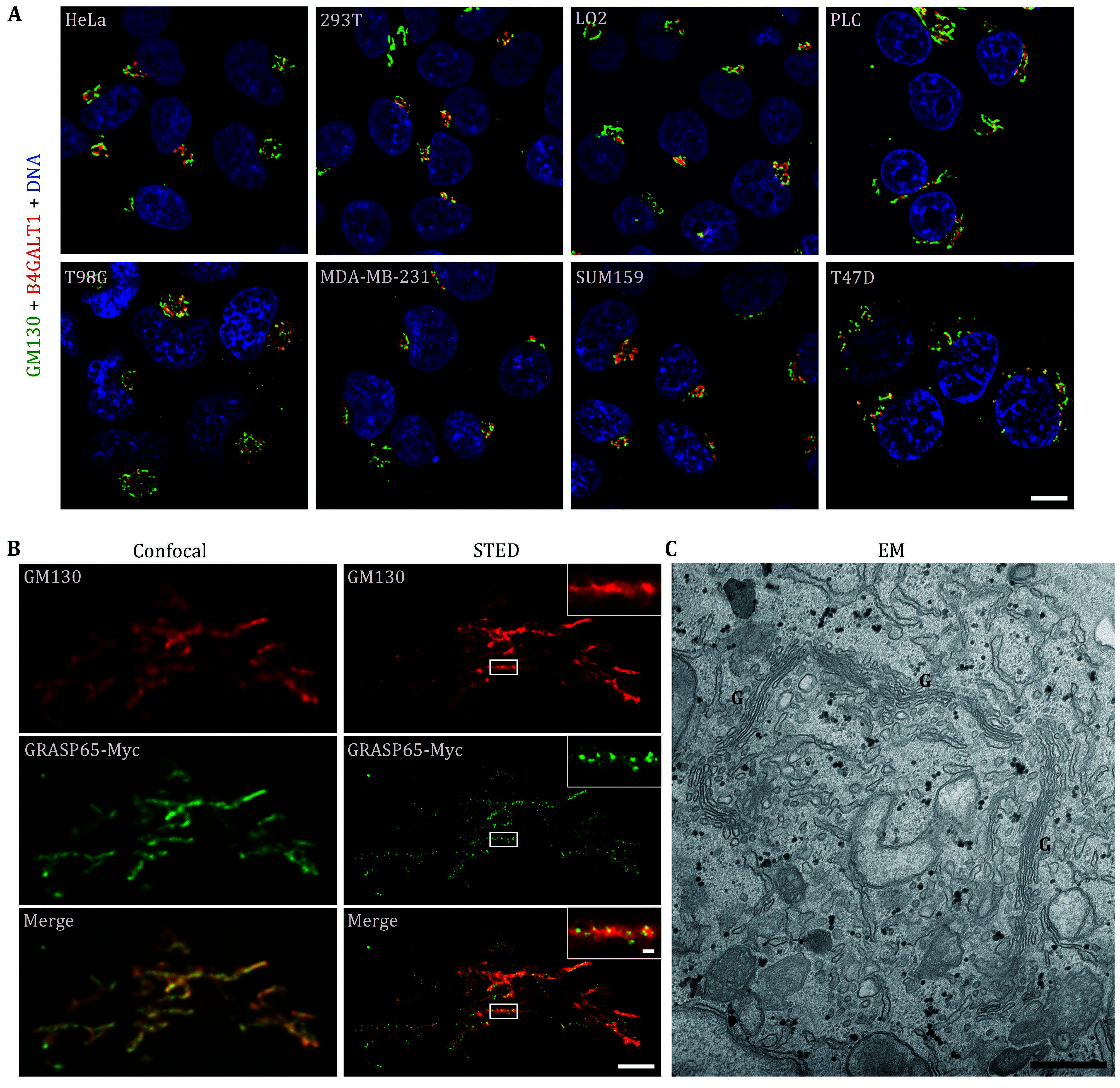
Images of the Golgi apparatus under different microscopy techniques. **A** Representative confocal microscopy images of the indicated cells demonstrated the variable morphology of the Golgi in different cell types. The cells were fixed and subsequently stained with antibodies against GM130 (a *cis-*Golgi marker, green) and B4GALT1 (a *trans-*Golgi marker, red). Scale bar, 10 μm. **B** Representative images obtained from both confocal and stimulated emission depletion (STED) microscopy show the unique distribution of Golgi proteins within HeLa cells. The cells were transfected with GRASP65-Myc and stained with antibodies for GM130 (red) and Myc (green). Scale bar, 2 μm, 200 nm (inserted panel). **C** Representative images from electron microscopy (EM) highlighted the ultrastructure of the Golgi apparatus within HeLa cells, denoted by "G". Scale bar, 500 nm

We utilized immunofluorescence microscopy to investigate the localization and morphology of the Golgi in various commonly used cell lines (Fig. 1A). Our observations revealed significant variations in Golgi structure among these cell lines. In HeLa cells, a compact perinuclear Golgi structure was consistently observed, typically considered representative of a normal Golgi architecture. Similarly, HEK293T and LO2 cells exhibited similar characteristics with compact perinuclear Golgi structures. However, in the liver cancer cell line PLC, the Golgi was relatively larger in area while maintaining its structural integrity. In contrast, the glioma cell line T98G exhibited a disrupted and fragmented Golgi structure, despite its perinuclear location, indicative of an abnormal phenotype. Although all breast cancer cell lines, MDA-MB-231 and SUM159, showed a compact structure, the Golgi in T47D cells exhibited obvious fragmentation, likely attributed to distinct genetic backgrounds present in these cell lines (Fig. 1A).

One common characteristic of cancer is the abnormal morphology of the Golgi apparatus. Although various morphological alterations of the Golgi have been observed in cancer cells, it is still unclear to what extent the changes in Golgi morphology can reflect the progression of malignant tumors. Furthermore, there are some cancer cell lines in which the Golgi morphology is indistinguishable from non-cancer cells, such as HeLa cervical cancer cells (Petrosyan 2015). Therefore, the precise features of the Golgi in cancer cells remain a subject of controversy.

In research, cell lines with relatively normal Golgi morphology are often selected for further study of Golgi structure and function. This allows for easily determining whether the targeted protein affects Golgi structure and causes Golgi fragmentation after gene knockout. Additionally, it is crucial to maintain normal cultural conditions to ensure the integrity of the Golgi. For example, amino acid starvation can induce autophagy, subsequently leading to Golgi fragmentation (Zhang *et al.* 2019b).

## OBSERVE GOLGI MORPHOLOGY USING VARIOUS MICROSCOPY TECHNIQUES

The Golgi is a delicate and relatively small cellular structure. Golgi cisternae are characterized as flattened, curved membranous structures, with each cisterna typically measuring between 0.5 to 1.0 μm in diameter and 20 to 50 nm in thickness (Tachikawa and Mochizuki 2017). It is important to note that the precise size of the Golgi cisterna may vary depending on factors such as the specific cell type, experimental conditions, and developmental stage. While the immunofluorescence microscopy, typically confocal microscopy, allows for the observation of the Golgi’s location within the cell, the internal structure of the Golgi apparatus cannot be well distinguished.

Fortunately, the availability of super-resolution microscopy has significantly addressed the resolution issue. GM130 and GRASP65 are both markers for the *cis-*Golgi. Under normal confocal microscopy, it was difficult to distinguish their differences because these two proteins overlapped well, creating an intertwined appearance (Fig. 1B, left panel). However, with STED (Stimulated Emission Depletion) microscopy, the same structure clearly revealed that GM130 was uniformly distributed on the Golgi apparatus, while GRASP65 existed in a punctate form (Fig. 1B, right panel). Hence, if the aim is to determine a protein's localization on the Golgi apparatus, super-resolution microscopy is the preferred choice.

Immunofluorescence is effective in illustrating the relative position of the Golgi within cells or between Golgi proteins. However, achieving a precise view of the Golgi morphology needs the use of a higher resolution electron microscope (EM). In fact, despite the initial discovery of the Golgi apparatus in 1898, its true nature was ultimately elucidated through EM. This technique revealed the distinctive stack structure that differs from other membranous structures. Under EM, it becomes evident that multiple Golgi stacks are laterally connected, forming what is known as a Golgi ribbon (Fig. 1C).

In general, a combination of immunofluorescence microscopy and EM is commonly employed to assess morphological alterations of the Golgi following the knockout of a target gene. Initially, immunofluorescence microscopy can be utilized to observe and determine if the encoded protein co-localizes with the known Golgi markers. If a co-localization is identified, subsequent analyses involving gene knockout or editing along with a comprehensive application of super-resolution and EM can be employed. This combined approach facilitates a thorough investigation into the effect of the target protein on the structure of the Golgi apparatus.

## DETERMINE THE PRECISE LOCALIZATION OF A TARGET PROTEIN ON THE GOLGI

Once we have identified the co-localization of target proteins and Golgi markers, the subsequent crucial step is to determine the specific cisternae on the Golgi where the proteins are located. Individual cisternae accumulate distinct structural proteins and enzymes that are essential for maintaining the precise function of the Golgi. For instance, different Golgins localize to specific regions of the Golgi and serve vesicle tethering at their respective positions (Table 1). GM130 and Golgin-160 are on the *cis*-Golgi, mainly tethering vesicles from ER. Golgin-84 resides at the edges of the *medial-*Golgi cisternae, mainly tethering vesicles involved in intra-Golgi transport. Golgin-245, Golgin-97, and GCC88 are located on the *trans-*Golgi, mainly receiving vesicles from the endocytic pathway (Muschalik and Munro 2018).

Various glycosylation enzymes are strategically distributed in different cisternae to ensure the sequential glycosylation modification of proteins (Kellokumpu *et al.* 2016). For instance, MAN1A1, an enzyme localized in the *cis-*Golgi, catalyzes the removal of one mannose residue from N-linked oligosaccharides during their maturation in the ER and Golgi. In contrast, ST6GAL1, an enzyme localized in the *trans-*Golgi, adds a sialic acid residue to the terminal galactose residue of glycoproteins and glycolipids.

**Figure 2:**
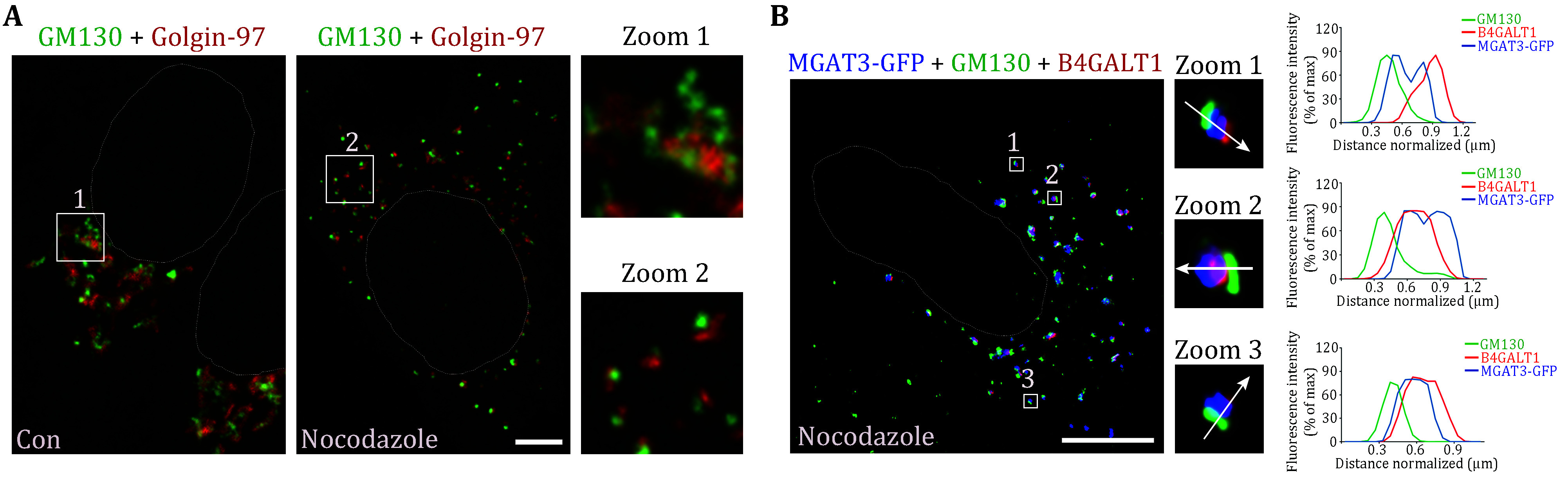
Localization of MGAT3 on the Golgi apparatus. **A** Representative confocal microscopy images of HeLa cells treated without (Con) or with nocodazole (0.5 μg/mL, 2 h) demonstrated Golgi ribbon and Golgi stacks respectively. HeLa cells were fixed and labeled with anti-GM130 (a *cis-*Golgi marker, green) and anti-Golgin-97 (a *trans-*Golgi marker, red) antibodies. The circled area indicated the nuclear area. Zoom: enlarged view of the Golgi area. Scale bar, 5 μm. **B** Representative confocal microscopy images of nocodazole-treated HeLa cells demonstrated the localization of MGAT3-GFP between the *cis*-Golgi and trans-Golgi. HeLa cells were transfected with MGAT3-GFP (blue in the image) and subsequently treated with nocodazole (0.5 μg/mL, 2 h). The cells were then labeled with anti-GM130 antibodies (green) and anti-B4GALT1 (a *trans-*Golgi marker, red) antibodies. The circled area indicates the nuclear area. Zoom: enlarged view of Golgi ministacks. White arrow across the stack was used for line-scan analyses and the distribution of fluorescence intensity of markers was quantified and normalized to their respective peak values. Scale bar, 10 μm

Determining the relative positions of two proteins within the Golgi can be challenging due to the distance between cisternae, measuring about 11 nm (Zhang and Wang 2015). However, if the Golgi ribbon is disassembled into smaller Golgi stacks, it becomes more feasible to observe the distinct locations of these proteins (Fig. 2A). This is usually accomplished by introducing nocodazole into cells, effectively disrupting microtubule polymerization, leading to the dispersal of Golgi stacks throughout the cytoplasm (Tang *et al.* 2011).

To precisely determine the localization of a target protein within the Golgi apparatus, nocodazole treatment to disperse the Golgi into mini stacks can be employed first. Subsequently, by co-labeling the target protein with the *cis-*Golgi marker GM130 and the *trans-*Golgi marker B4GALT1, it becomes possible to discern if the target protein is closer to the *cis*- or *trans*-side. In our experiment, the target protein of interest, MGAT3, which is responsible for catalyzing the transfer of an N-acetylglucosamine residue to the beta-1,4-linked mannose on N-linked glycoproteins, was observed to be in closer proximity to the *trans-*Golgi (Fig. 2B). In fact, Immuno-EM would be a preferred method for a more precise determination of the target protein's location, although it is technically challenging and requires the availability of high-quality antibodies.

## QUANTIFY THE GOLGI CISTERNAL LENGTH AND NUMBER

**Figure 3:**
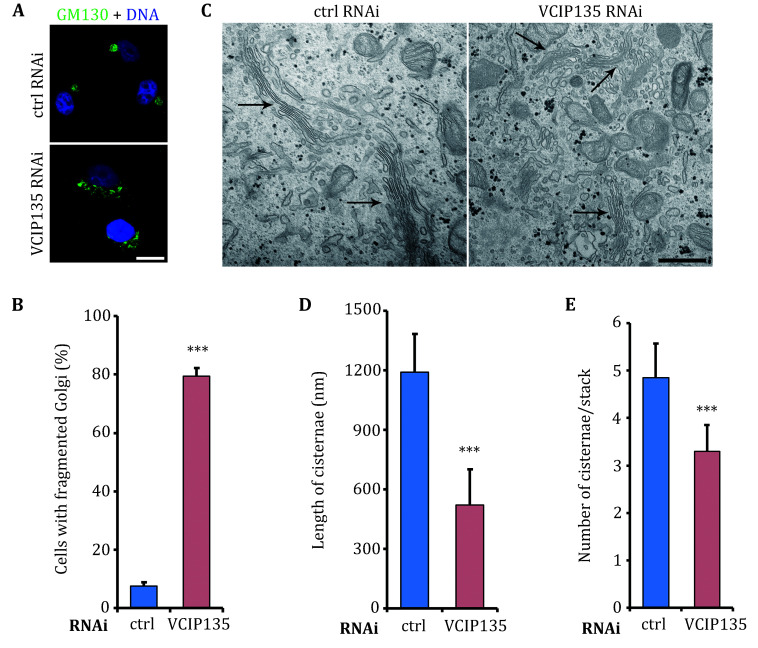
Depletion of VCIP135 leads to Golgi fragmentation. **A** Representative fluorescence images of HeLa cells transfected with control or VCIP135 RNAi, respectively. The cells were immunostained with GM130. Scale bar, 20 μm. **B** Quantification of the cells in Panel A, presented as the percentage of cells with fragmented Golgi. This analysis was carried out in three sets of independent experiments, where more than 200 cells were counted in each experiment. **C** Representative electron micrographs of cells treated with control and VCIP135 RNAi, respectively. The Golgi stacks were indicated by arrows. It is noteworthy that the length of the cisternae in VCIP135-depleted cells was reduced. Scale bar, 500 nm. **D**,**E** Quantification of the EM images as in Panel C, from three independent experiments. Twenty different cells were measured in each set of experiments. The results were presented as means ± SEM, ***P < 0.001, Student's *t*-test. This figure is adapted and modified from Zhang *et al.* (2014)

Once the subcellular localization of the target protein has been confirmed, the next step typically involves investigating how the gene knockout affects both the structure and function of the Golgi. To observe any structural alterations, immunofluorescence can be employed to examine the alteration of Golgi morphology. For instance, in the case of the Golgi-localized deubiquitinating enzyme VCIP135, knockdown experiments resulted in remarkable fragmentation of the Golgi (Fig. 3A) (Zhang *et al.* 2014). Furthermore, the significance of this effect can be assessed by statistically comparing the proportion of fragmented Golgi between control and knockdown cell lines (Fig. 3B).

To obtain precise insights into how the structure of the Golgi is specifically influenced, it is recommended to perform EM analysis after the gene knockout. It became readily apparent that, compared to normal cells, the Golgi stacks in VCIP135 knockdown cells appeared smaller in size (Fig. 3C). To provide a more accurate characterization of this impact, two main indicators are commonly measured. Firstly, the length of Golgi cisternae was assessed by measuring the longest cisternae within the stack (Fig. 3D). Secondly, the number of Golgi cisternae was determined by counting the stack with the largest number of cisternae in the cell (Fig. 3E). These measurements indicated that VCIP135 knockdown reduced both length and the number of Golgi stack (Zhang *et al.* 2014).

Therefore, based on the analysis of immunofluorescence and electron microscopy images, as well as the statistical results obtained from the gene knockdown experiments, a clear conclusion can be made that VCIP135 plays a vital role in the assembly of the Golgi apparatus.

## CHARACTERIZE CARGO GLYCOSYLATION BY LECTIN STAINING

Glycosylation is the most complex and diverse post-translational modification. It requires the coordinated action of various glycosyltransferases, glycosidases, nucleotide sugar transport proteins, and substrates to add monosaccharides or sugar chains to proteins or lipids (Schjoldager *et al.* 2020). Glycosylation plays important regulatory roles in cell signaling, intercellular communication, and the interactions between cells and the extracellular matrix (Reily *et al.* 2019). Incorrect glycosylation modifications have the potential to disrupt the proper function of glycoproteins, thereby giving rise to consequences such as cancer cell migration and immune escape (Pinho and Reis 2015). Therefore, in-depth studies are required to unravel the underlying regulatory mechanisms governing glycosylation.

Protein glycosylation is mainly categorized into two major types: N-linked glycosylation and O-linked glycosylation. The process of N-linked glycosylation starts within the rough ER and completes in the Golgi, whereas O-linked glycosylation predominantly occurs within the Golgi. Proper glycosylation modification relies on the normal structure of the Golgi. Notably, the disruption of Golgi structure in cancer cells often coincides with the defects of glycosylation. Therefore, when studying the impact of target genes on the Golgi structure, the influence of target genes on glycosylation is also examined.

**Figure 4:**
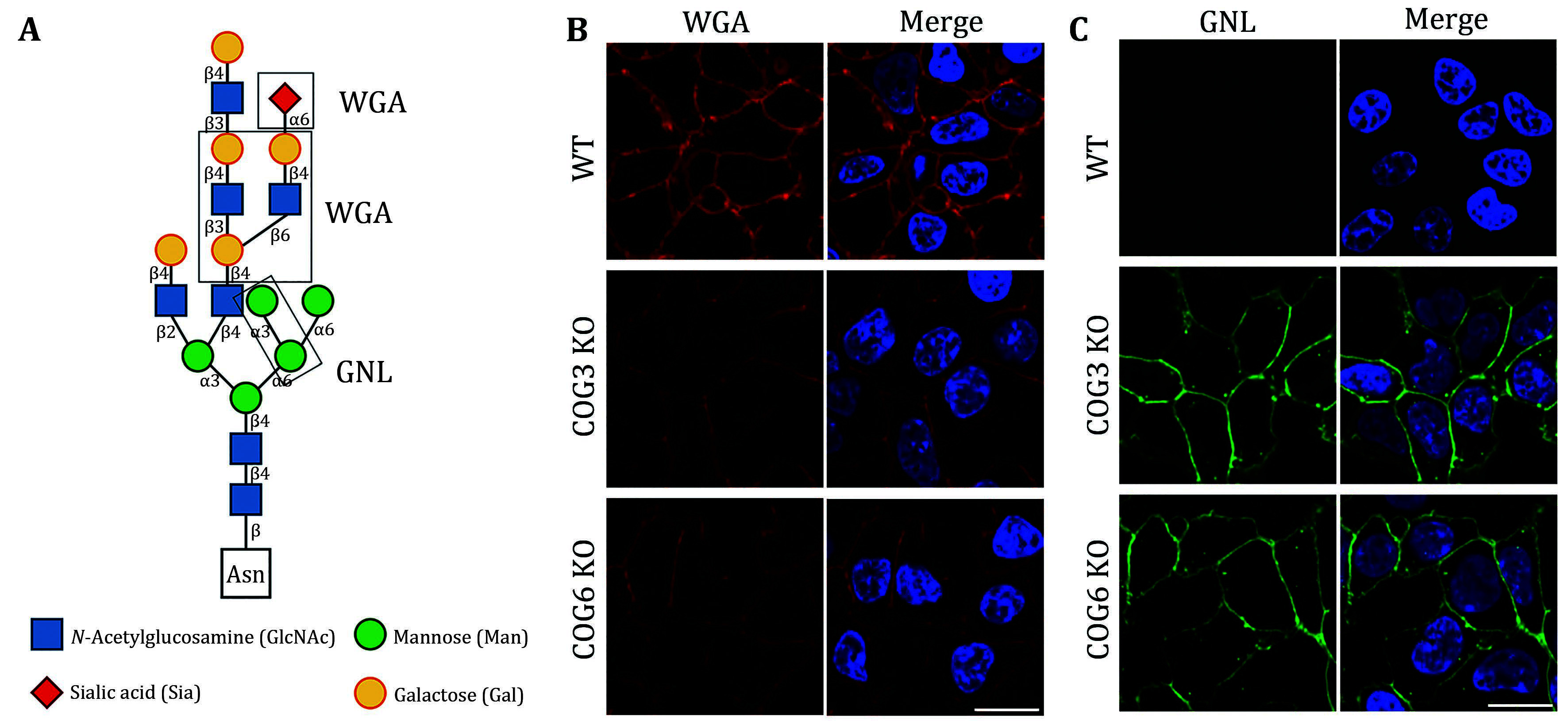
Lectin staining was conducted to investigate the alterations in the glycosylation patterns of plasma membrane glycoconjugates. **A** Annotation of the graph indicates that both WGA and GNL lectins selectively bind to specific N-glycan motifs: WGA has an affinity for terminal Sialic Acid and β1-4-linked Galactose that is branched to both a β1-3-linked N-Acetylglucosamine and a β1-6-linked N-Acetylglucosamine, whereas GNL preferentially binds to terminal α1-3-linked Mannose residues. **B** Analysis of plasma membrane staining of wild-type (WT) 293T cells and those with COG3 or COG6 knockout (COG3 KO or COG6 KO) cells with WGA-Rhodamine (red). The nuclei were stained with DAPI (blue). Scale bar, 20 µm. The WGA staining revealed a marked reduction in lectin binding in comparison to 293T WT cells, suggesting a deficiency in terminal sialylation of proteins. **C** A similar investigative approach was taken for plasma membrane staining of 293T WT and COG3 KO or COG6 KO cells using GNL-Fluorescein (green). Scale bar, 20 µm. The enhanced GNL binding in the plasma membrane glycoconjugates of COG3 KO and COG6 KO cells compared to 293T WT cells, indicated an increased presence of terminal α1-3-linked Mannose in these structures

There are typically two commonly used methods for the detection of glycosylation. The first method involves glycan mass spectrometry, which enables the detection of the precise sugar composition. This method, however, tends to be intricate and costly. The second method is via lectin staining, which identifies specific sugar structures (Table 2). Typically, a combination of various lectins is used to comprehensively evaluate the glycosylation pattern of cell surface proteins (Ren and Fujita 2023). For example, WGA recognizes terminal sialic acid, while GNL recognizes terminal α1-3-linked mannose (Fig. 4A, Table 2).

**Table 2:** Commonly used lectins in Golgi-related study

Name	Abbreviation	Recognition	Recognition	Publications
*Galanthus nivalus* lectin	GNL	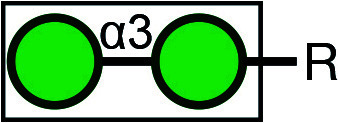	Terminal α1-3-linked Mannose	Bailey Blackburn *et al.* (2016)
*Helix pomatia* agglutinin	HPA	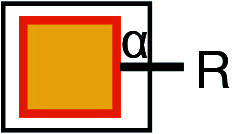	α-linked terminal GalNAc	Sumya *et al.* (2021)
*Wheat germ* agglutinin	WGA	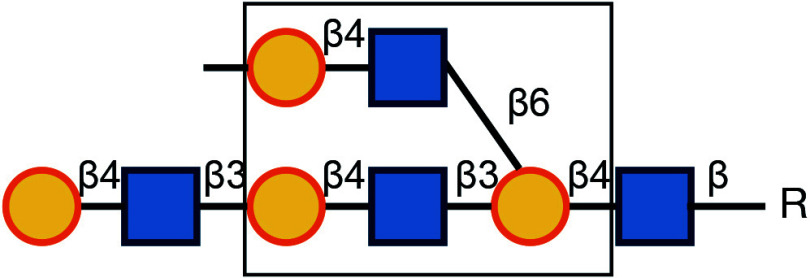	β1-4-linked Gal branched to one β1-3-linked GlcNAC and one β1-6-linked GlcNAC, each bound to a β1-4-linked Gal (core of glycan)	Pokrovskaya *et al.* (2011), Xiang *et al.* (2013)
		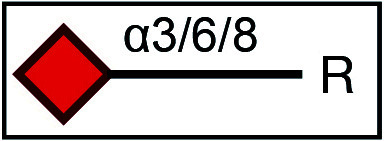	α2-3-, -6-, -8- linked terminal Sialic Acid	
		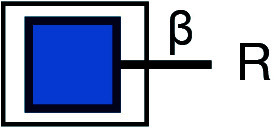	β-linked terminal GlcNAc	
*Ricinus Communis* agglutininI	RCA-I	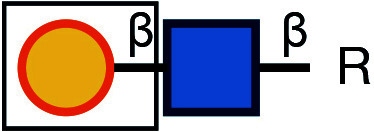	β-linked terminal Gal	Bailey Blackburn *et al.* (2016)
Concanavalin A	ConA	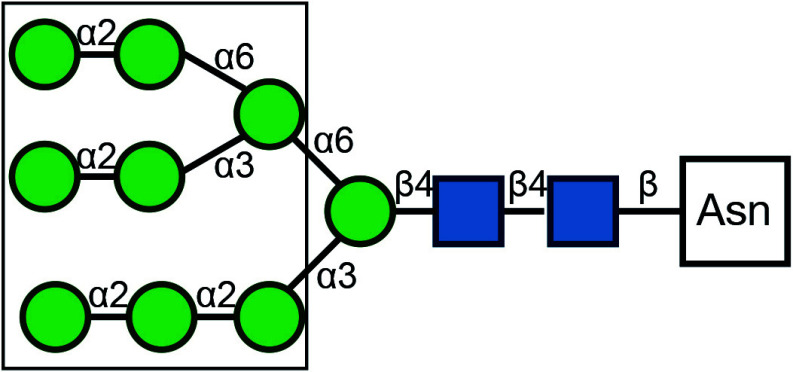	Oligomannose-type N-glycan	Bui *et al.* (2023)
		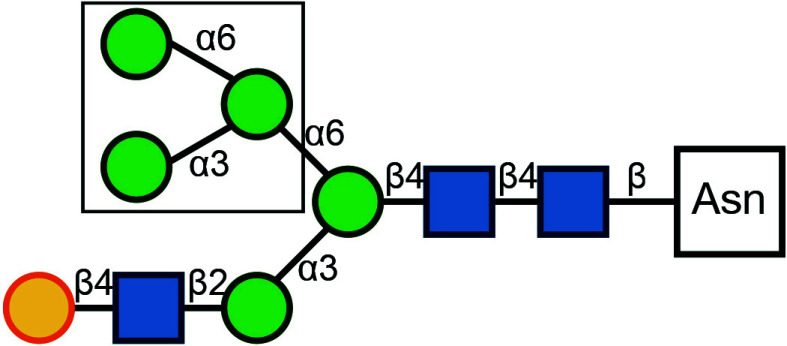	Hybrid-type N-glycan	
		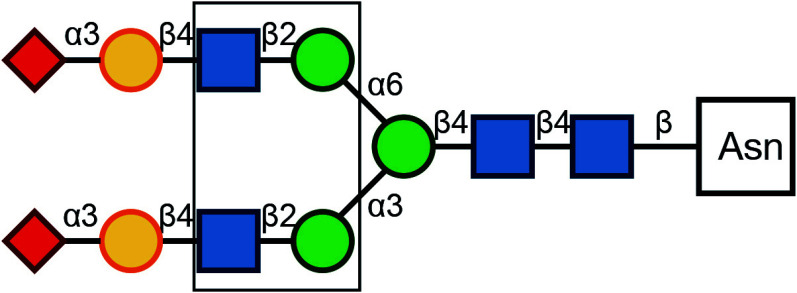	Biantennary complex-type N-glycan but not tri-and tetraantennary complex-type N-glycan	
Peanut Agglutinin	PNA	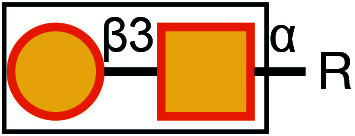	β1-3-linked terminal Gal to α-linked GalNAc	Pokrovskaya *et al.* (2011)
*Griffonia simplicifolia* lectin II	GSL-II	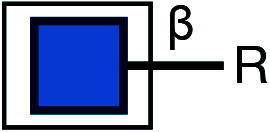	β-linked terminal GlcNAc	Pokrovskaya *et al.* (2011)
		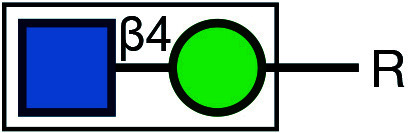	β1-4-linked terminal GlcNAc (to core Man)	
*Maackia amurensis* leukoagglutinin	MAL	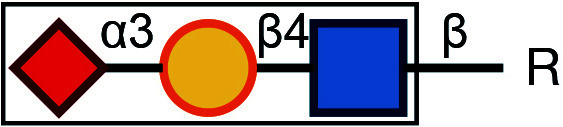	α2-3-linked terminal SialicAcid to β1-4-linked Gal to β-linked core GlcNAC	Xiang *et al.* (2013)
Symbol Key: 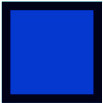 *N*-Acetylglucosamine (GlcNAc); 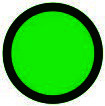 Mannose (Man); 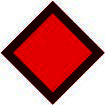 Sialic acid (Sia); 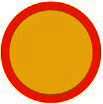 Galactose (Gal); 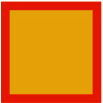 *N*-Acetylgalactosamine (GalNAc)				

The conserved oligomeric Golgi complex (COG Complex) is an octameric complex that can be divided into two subcomplexes: Lobe A (COG1-4) and Lobe B (COG5-8). The complex mediates the retrograde transport and correct localization of glycosyltransferases, ensuring the ordered glycosylation modification of proteins. Knockout of COG subunits affected the localization of glycosylation enzymes, resulting in defective glycosylation (D'Souza *et al.* 2020). In this study, knockout of COG3 or COG6 weakened the staining of WGA, indicating a deficiency in the addition of the terminal sialic acid in N-linked glycosylation (Fig. 4B), while the staining of GNL was significantly increased, indicating the production of more incomplete glycans (Fig. 4C). These findings suggested that COG3 and COG6 played a regulatory role in Golgi glycosylation. Therefore, gene knockout combined with lectin staining serves as a valuable approach to investigating the potential influence of target proteins on glycosylation modification.

## SUMMARY AND PERSPECTIVES

To explore whether a target protein is located in the Golgi and influences its structure and function, it is critical to select appropriate cell lines for experiments. Typically, multiple cell lines with relatively normal Golgi apparatus morphology are used for mutual validation. The detection of the target protein in the Golgi can be achieved through immunofluorescence microscopy, super-resolution microscopy, and immuno-EM using antibodies specific to the target protein. By comparing the relative positions of the target protein with the *cis-*Golgi and *trans-*Golgi markers, their specific locations within the Golgi can be determined. Additionally, upon knocking out the target gene, the impact on the Golgi structure can be evaluated by measuring the length and number of Golgi cisternae. Furthermore, lectin staining can be employed to assess whether the target protein has an impact on Golgi-regulated glycosylation modifications. In summary, the localization and function of the target protein in the Golgi can be preliminarily determined through multiple microscopy techniques.

For a more in-depth investigation into the molecular mechanism of the target protein on the Golgi, biochemical experiments are necessary. One approach involves purifying the Golgi apparatus from cells or tissues followed by using Western blotting and mass spectrometry to determine whether the target protein is present in the Golgi (Fasimoye *et al.* 2023). Furthermore, the use of ultracentrifugation techniques enables the identification of the target protein's localization, such as whether it resides on the Golgi membrane or functions as peripheral Golgi membrane proteins.

There are several issues that demand more effort to address the following questions in the field of Golgi biology: first, what are the mechanism and specific functions of forming flat stack and ribbon structures in the Golgi of mammalian cells; second, how the abundant enzymes and substrates in the Golgi apparatus are precisely located and undergo orderly processing; third, how the Golgi apparatus, as a transport hub, interacts with other organelles under various conditions.

The isolation of pure Golgi membranes by biochemical approaches and precise localization of Golgi glycosyltransferases and substrates utilizing techniques like super-resolution microscopy would significantly contribute to unraveling the working model governing this organelle. At the same time, investigating the mechanisms underlying structural and functional alterations of the Golgi in physiological and pathological conditions will help us understand human diseases and provide a new theoretical basis for the diagnosis and treatment of diseases.

## EXPERIMENTAL SECTION

### Cell culture, transfection, and treatment

All the cell lines were cultured in a 5% CO2 incubator at 37 °C. HeLa, HEK293T, HepG2, T98G, MDA-MB-231, T47D, and PLC cells were grown in DMEM medium supplemented with 10% donor bovine serum. LO2 and SUM159 cells were cultured in RPMI-1640 medium supplemented with 10% donor bovine serum. Both mediums were also supplemented with 100 U/mL penicillin and 100 μg/mL streptomycin.

For COG3 or COG6 knockout (KO) 293T cells, the target sequence for COG3 (GGATCGGAGACCGGACACGA) or COG6 (GTTGTTGGGCATATCACTGA) was cloned into the lentiCRISPR v2 vector. The resulting plasmids were then transfected into 293T cells for 24 h. Following transfection, the cells underwent selection with 1.5 μg/mL puromycin for a period of four to five days, after which individual cell clones were isolated using a 96-well plate. KO cells were subsequently identified through DNA sequencing and immunoblot analysis.

Polyethylenimine (PEI) was used to transiently transfect pEGFP-N1-MGAT3. Briefly, cells were cultured until they reached approximately 70% confluence. A master transfection reagent mix was prepared for a 10-cm petri dish, consisting of 1 mL DMEM, 30 µL PEI stock (1 mg/mL, pH 7.0), and 10 µg of plasmids. The mix was then incubated at room temperature for 10–15 min. Once the mix was ready, it was slowly added to the cells. Cells were collected for further analysis 18–24 h after transfection.

Lipofectamine RNAiMAX was utilized to transfect HeLa cells for VCIP135 knockdown (KD) following the manufacturer's instructions. Briefly, cells were cultured until they reached about 70% confluence. Subsequently, control non-specific RNAi oligo or specific RNAi oligo targeting human VCIP135 (5’-GCAAATGTGGTTGTGGATT-3’) was diluted in an Opti-MEM medium (Zhang *et al.* 2014). Similarly, the Lipofectamine RNAiMAX reagent was diluted separately. These solutions were then mixed at a 1:1 ratio. The mixture was incubated for another 5 min and was then gently added to the culture media of the HeLa cells. The cells were transfected for 72 h before subsequent assays.

To apply nocodazole treatment, the pEGFP-N1-MGAT3 transfected cells were exposed to 0.5 μg/mL μnocodazole for 2 h, after which immunofluorescence microscopy was performed.

### Immunofluorescence of cultured cells

Indicated cells were cultured on sterilized coverslips to semi-confluence and then washed with phosphate-buffered saline (PBS) at room temperature. After removing the PBS, the cells were fixed with 4% paraformaldehyde (PFA) in PBS for 15 min. The PFA was then aspirated, and the cells were quenched with 50 mmol/L NH_4_Cl in PBS for 10 min. Following quenching, the NH_4_Cl solution was discarded, and the cells were permeabilized with a 0.3% Triton X-100 (*v*/*v*) solution in PBS for 10 min, which was prepared by diluting a 10% Triton stock solution with PBS. The cells were then washed twice with PBS. The primary antibodies, anti-GM130 and anti-B4GALT1 in Fig. 1A, prepared in 3% bovine serum albumin (BSA) in PBS, were then applied to the fixed cells, and they were incubated overnight at 4 °C. After the incubation with the primary antibodies, the cells were washed three times with PBS on a shaker, followed by incubation with a secondary antibody solution (prepared in 3% BSA) conjugated with fluorescent labels such as FITC (green) and TRITC (red), for 30 min to 1 h at room temperature. The cells were then washed three more times with PBS on a shaker, and the coverslips were mounted onto microscope slides using an antifade mounting medium containing DAPI and left to air dry. The specimens were finally examined via Andor confocal spinning disk on a Nikon inverted microscope Eclipse Ti-E.

### Confocal and STED microscopy

HeLa cells were transfected with GRASP65-Myc and then stained with anti-Myc and anti-GM130 primary antibodies. For STED (Stimulated Emission Depletion) microscopy, it was necessary to use a secondary antibody fluorescent dye that is highly photostable and can be efficiently used with an STED laser wavelength between 750–800 nm. In this study, Abberior STAR ORANGE and Abberior STAR RED secondary antibodies were employed. Confocal and STED images in Fig. 1B were acquired using the Abberior STEDYCON fluorescence microscope, which is built on a motorized inverted microscope IX83 (Olympus UPlanXAPO 100x, NA1.45, Tokyo, Japan) from Abberior Instruments GmbH.

### Electron microscopy

HeLa cells were transfected with control and VCIP135 siRNA for 72 h (Zhang *et al.* 2014), and then seeded into six-well dishes. After another 24-h culture, cells were processed for the Epon embedding procedure. For fixation, DMEM medium containing 20 mmol/L Hepes (pH 7.4) was warmed up to 37 °C, followed by the addition of 25% glutaraldehyde to achieve a final concentration of 2% immediately before fixation. The growth medium was then removed from the cells, and the fixative was directly applied to the dish to ensure complete coverage. The dish was left at room temperature for 30 min or overnight at 4 °C if needed. The cells were washed twice with 0.1 mol/L sodium cacodylate and transferred to the chemical hood for subsequent steps. They were post-fixed for 1 h on ice with a solution containing 1% (*w*/*v*) osmium tetroxide (OsO4), 1.5% potassium ferricyanide, and 0.1 mol/L sodium cacodylate (pH 7.4), covering the entire dish.

For staining, the cells were washed three times with 50 mmol/L Maleate buffer (pH 5.2), followed by staining with 2% uranyl acetate (UA) in 50 mmol/L sodium Maleate (pH 5.2) for 1 h. The cells were washed three times with H_2_O, scraped off the dish with H_2_O, and divided into two or three Eppendorf tubes, ensuring at least one tube had an optimally sized pellet. The cells were centrifuged at 15,000 r/min for 3 min to compact the pellet. Dehydration was achieved using a series of ethanol solutions, namely 500 μL of 50% (2 × 5 min), 70% (2 × 5 min), 90% (2 × 5 min), and 100% ethanol (3 × 15 min). Afterward, the pellets were treated with 500 μL propylene oxide, repeated three times for 15 min with a closed lid.

The cells underwent infiltration with a 1:1 mixture of Epon-mix (composed of 14.5 g Embed 812, 10.5 g NMA, and 5.0 g DDSA mixed thoroughly until no streaks remained; 0.54 g DMP-30 was added and mixed immediately before use) and propylene oxide for 1 h with a closed lid, extending the duration for larger pellets. This infiltration step was then repeated for 1 h with an open lid. The cells were infiltrated with pure Epon-mix and rotated for 1 h, repeated three times with an open lid, or left overnight as required. Next, 500 μL of Epon-mix was added to the tubes and polymerized at 60 °C overnight (Tang *et al.* 2010).

Ultrathin sections of 60 nm were cut with a Reichert Ultracut S (Leica) using a diamond knife and mounted onto Formvar-coated nickel grids. The sections were stained with 2% UA for 5 min at room temperature, shielded to prevent oxidation, and washed thoroughly with H_2_O. They were then contrasted with 3% lead citrate for 5 min, followed by a rinse with H_2_O and allowed to dry. Finally, the grids were examined using a JEOL transmission electron microscope. Images were captured from over 20 cells at a magnification of 11,000×. These images were taken from the perinuclear region of the cell, where Golgi membranes are typically concentrated.

### Quantification of length and numbers of Golgi cisternae

A cisterna within the Golgi cluster was defined as a membrane-bound structure with a length that is at least four times its width, while the width itself doesn't exceed 60 nm. A stack, on the other hand, refers to multiple flattened cisternae that are piled up together. Using ImageJ, the length of the longest cisternae within a Golgi stack was measured, while the number of cisternae layers in the Golgi stack with the highest quantity was counted as the number of cisternae per stack in a cell (Zhang *et al.* 2019a). In the experiments comparing the Golgi in HeLa WT and VCIP135 KD cells, the abnormalities in Golgi stack formation were characterized by (1) a decreased number of cisternae per stack, and (2) a reduced length of the cisternae within each stack (Zhang *et al.* 2014).

### Lectin staining

Human embryonic kidney 293T (HEK293T) cells, both wild-type (WT) and with COG3 or COG6 gene knockouts (KO), were seeded onto sterilized glass coverslips and expanded until they reached 70%–80% confluency. After media removal, the cells were washed with PBS at room temperature to eradicate any cellular debris and remnants of the growth medium. The cells were then fixed with a freshly prepared 1% (*w*/*v*) solution of PFA in PBS for 15 min (Bailey Blackburn *et al.* 2016). The fixation was succeeded by a thorough PBS wash, and then the cells proceeded to be incubated under blocking conditions utilizing a 1% (*w*/*v*) bovine serum albumin (BSA) solution in PBS for 10 min to prevent non-specific binding.

In preparation for lectin binding assays, the cells were incubated with fluorescently labeled lectins: fluorescein-conjugated *Galanthus nivalis* lectin (GNL-Fluorescein) at 20 μg/mL and rhodamine-conjugated Wheat Germ Agglutinin (WGA-Rhodamine) at 2.5 μg/mL, and both diluted in 1% BSA/PBS, shielded from light exposure, at room temperature for 30 to 60 min. After lectin exposure, the samples were rinsed five times with PBS, including a 2-min agitation on a shaker between each wash to thoroughly remove unbound reagents. Finally, the coverslips were mounted on microscope slides using an Antifade Mounting Medium supplemented with DAPI for nuclear staining and were left to air-dry. The images were obtained through an Andor confocal spinning disk apparatus integrated with a Nikon Eclipse Ti-E inverted microscope system.

## Conflict of interest

Yusheng Xing, Yannan Jian, Xiaodan Zhao, Yue Zhang, Zhenqian Zhang, Xing Zhang and Xiaoyan Zhang declare that they have no conflict of interest.
